# The Use of Critical Race Theory to Inform Culturally Adapted Recruitment Strategies for Community-Based Research

**DOI:** 10.1093/geroni/igab046.1868

**Published:** 2021-12-17

**Authors:** Lauren Parker, Joseph Gaugler, Laura Gitlin

**Affiliations:** 1 Johns Hopkins Bloomberg School of Public Health, Baltimore, Maryland, United States; 2 University of Minnesota, Minneapolis, Minnesota, United States; 3 Drexel University, College of Nursing and Health Professions, Drexel University, Pennsylvania, United States

## Abstract

Black/African American participation in non-pharmacological randomized trials and community-based research on Alzheimer’s Disease and related dementias (ADRD) remains low. This presentation introduces Critical Race Theory (CRT), its tenets and how it can be used in tandem with national strategies to encourage participation of Black/African Americans in ADRD research. CRT is a transdisciplinary methodology that draws on anti-racist tenets to study and transform the relationships among race, racism, and power. We describe how CRT constructs (e.g., race consciousness, counter-storytelling) can be used to inform recruitment strategies to enroll Black/African American ADRD caregivers into community-based research by drawing upon two ongoing studies: a randomized trial providing caregiver support through Adult Day Services (ADS) and the evaluation of impact of ADS on stress levels of Black/African American using biomarker measures. In the spirit of CRT this presentation is a call for action and transformation of dementia care research to actively include Black/African Americans.

